# The Role of FNDC5/Irisin in Cardiovascular Disease

**DOI:** 10.3390/cells13030277

**Published:** 2024-02-02

**Authors:** Maciej Grzeszczuk, Piotr Dzięgiel, Katarzyna Nowińska

**Affiliations:** 1 Division of Histology and Embryology, Department of Human Morphology and Embryology, Wroclaw Medical University, 50-368 Wroclaw, Poland; maciej.grzeszczuk@student.umw.edu.pl (M.G.); piotr.dziegiel@umw.edu.pl (P.D.); 2 Department of Human Biology, Faculty of Physiotherapy, Wroclaw University of Health and Sport Sciences, 51-612 Wroclaw, Poland

**Keywords:** irisin, fibronectin type III domain-containing protein 5, *FNDC5*, myocardial infarction

## Abstract

Disorders of cardiomyocyte metabolism play a crucial role in many cardiovascular diseases, such as myocardial infarction, heart failure and ischemia–reperfusion injury. In myocardial infarction, cardiomyocyte metabolism is regulated by mitochondrial changes and biogenesis, which allows energy homeostasis. There are many proteins in cells that regulate and control metabolic processes. One of them is irisin (Ir), which is released from the transmembrane protein FNDC5. Initial studies indicated that Ir is a myokine secreted mainly by skeletal muscles. Further studies showed that Ir was also present in various tissues. However, its highest levels were observed in cardiomyocytes. Ir is responsible for many processes, including the conversion of white adipose tissue (WAT) to brown adipose tissue (BAT) by increasing the expression of thermogenin (UCP1). In addition, Ir affects mitochondrial biogenesis. Therefore, the levels of FNDC5/Ir in the blood and myocardium may be important in cardiovascular disease. This review discusses the current knowledge about the role of FNDC5/Ir in cardiovascular disease.

## 1. Introduction

In 2012, Boström et al. discovered a new protein and named it Irisin (Ir), after the Greek goddess Iris, who was a messenger conveying the will of the gods to humans. The name of the protein is related to the communication between muscles and other tissues, such as fat and bone tissues. Research on Ir has indicated that it is commonly found in the human body—in the heart, brain, skeletal muscle, liver, kidney, lung, spleen, skin, secretory glands, adipose tissue and myelin sheaths of nerve fibers [1,2,3,4,5]. Ir levels, however, vary across different tissues. The highest levels were reported in cardiomyocytes, while slightly lower concentrations were found in the brain and skeletal muscles. Further research indicated that higher Ir levels could also be found in various types of cancers, including stomach [6], bladder [7], lung [8], larynx [9], or breast cancers [10,11]. This suggests that Ir may have a role in altering the metabolism of cancer cells and may be useful in oncological diagnosis [12,13]. In addition, the presence of Ir released from cells was also observed in serum. Its serum levels are affected by obesity, cardiovascular disease, diabetes, gestational diabetes, non-alcoholic fatty liver disease, lipid metabolism disorders, metabolic bone disease and polycystic ovary syndrome [14].

### 1.1. The Structure of FNDC5/Irisin

Irisin is released from the transmembrane fibronectin type III domain-containing protein 5 (FNDC5), which is encoded by the *FNDC5* gene [15]. This gene is located on chromosome 1 at position 35.1 (1p35.1). In the human *FNDC5* gene, a mutation within the start codon is observed. In humans, ATA (isoleucine) is the start codon of *FNDC5* [16]. In turn, ATG (methionine) is the start codon in rats, mice, gibbons, gorillas and chimpanzees [17]. This may suggest higher expression of the *FNDC5* gene in animals than in humans. In addition, *FNDC5* gene expression is regulated by the proliferator-activated receptor gamma coactivator-1α (PGC1α) [18] and may also be regulated by the estrogen-related receptor alpha (ERRα). Both PGC1α and ERRα proteins may be related [13]. Nuclear PGC1α is a transcription factor involved in the regulation of *FNDC5* expression in various tissues [19]. Kim et al. [20] suggested that *FNDC5* gene transcription was epigenetically regulated by histone H3 acetylation and H3K27 methylation. Both acetylation and methylation downregulate *FNDC5* gene expression in cancer cells [21]. During embryonic development, the *FNDC5* gene is involved in cardiogenesis. The overexpression of *FNDC5* during cardiac differentiation leads to a significant increase in the expression of progenitor and cardiomyocyte markers, facilitating cardiogenesis [22]. The expression of *FNDC5* gradually increases during the differentiation of mouse embryonic stem cells (mESCs) into cardiac cells. The inhibition of *FNDC5* expression affects mitochondrial impairment in cardiomyocytes [23]. The PGC1α–FNDC5 pathway is responsible for it in the differentiation of mESC cardiomyocytes [24].

The expression of the *FNDC5* gene results in the formation of different Ir isoforms. Isoform 1 (Q8NAU1-1) is considered a canonical sequence. The other three isoforms (isoform 2 (Q8NAU1-2), isoform 3 (Q8NAU1-3) and isoform 4 (Q8NAU1-4)) have missing sequences of amino acids at positions 182–212, 1–75, 1–75, respectively [25]. Changes in the amino acid sequence result in different protein lengths. However, it is not known whether these changes affect the function of the protein and the physiology and pathology of the cardiovascular system.

**Figure 1:**
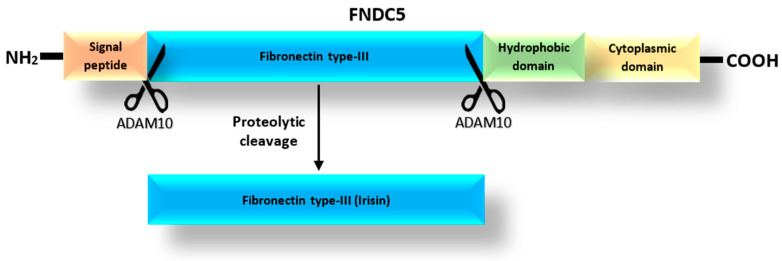
Structure of the FNDC5 protein based on the spectrometric analysis and the mechanism of Ir release as modified from Young et al. [26].

According to the UniProt database [25], the FNDC5 protein, which is a precursor of Ir [18], can exist as one of four isoforms with lengths of 212, 181, 153 and 137 amino acids. The prohormone FNDC5 consists of a signal peptide, the fibronectin type III-like domain, and the hydrophobic carboxyl-terminal domain (Figure 1) [26]. Irisin is released after the proteolytic cleavage of the extracytoplasmic part of the FNDC5 protein, which includes the fibronectin-type III domain [1]. The release of Ir is mediated by one of the proteases of the ADAM family, most likely the ADAM10 protease, as suggested by Yu et al. [27]. In turn, Schumacher et al. [28] observed that Ir released into the blood could combine to form dimers. Once dimerized, it has the potential to affect cells in an autocrine or paracrine manner. In addition, Kim et al. [29] found that Ir was a ligand for the αVβ5 integrin receptor in osteocytes and adipose tissues. They proposed a mechanism by which Ir binds to integrin αVβ5 after dimerization.

### 1.2. Signaling Pathways Related to FNDC5/Irisin in Heart and Cardiovascular Diseases

Major signaling pathways associated with Ir include the mitogen-activated protein kinase (MAPK) pathways involved in neuronal differentiation, the browning of white adipocytes and osteoblast proliferation and differentiation. Other pathways mediating FNDC5/Ir functions include AMPK, PI3K/AKT and STAT3/Snail [30]. It is thought that Ir may influence the protection of the heart and coronary microvasculature from damage through various signaling pathways.

Ir protects against cardiac damage induced by diabetic cardiomyopathy, cardiomyocyte mitochondrial damage and pyroptosis. The protective effect is achieved through the activation of mitochondrial ubiquitin ligase (MITOL) via the cGAS/STING pathway [31], which is involved in the pathophysiology of cardiovascular disease and risk factors via the innate immune pathway. The protective effect in cardiovascular disease involves the attenuation of inflammatory senescence [32].

In addition, Ir attenuates diabetic cardiomyopathy by activating αV/β5-AKT integrin signaling and reducing oxidative/nitrosative stress [33]. Ir protects cardiomyocytes from apoptosis, reactive oxygen species and inflammation through the AMPK/mTOR signaling pathway [34].

By activating the ERK1/2 pathway, Ir enhances the proliferation and migration of cardiac microvascular endothelial cells (CMECs), thereby improving cardiac microcirculation. In addition, Ir influences the reduction in oxidative stress in CMECs through the ERK1/2/Nrf2/HO-1 pathway [35]. Through the MEK1/2 pathway, KV channels and calcium-activated potassium channels (SK_Ca_ and BK_Ca_), Ir induces relaxation responses related to MEK1/2 pathway activity in intact and endothelium-denuded aortic vessels [36].

Ir affects the inactivation of the LOXL2 and TGFβ1/Smad2/3 signaling pathways, thereby inhibiting Ang II-induced atrial fibrosis and susceptibility to atrial fibrillation [37].

Ir may affect the regulation of vascular smooth muscle cell (VSMC) phenotype modulation through the STAT3 signaling pathway. It also protects against vascular calcification by activating autophagy and inhibiting pyroptosis in VSMCs via NLRP3 [38]. Additionally, it activates AMPK, reduces the expression of Drp1 and its translocation to mitochondria, and subsequently inhibits mitochondrial fission [39]. 

To date, the regulation and signaling associated with Ir have not been fully elucidated. Further research is warranted to clarify the relationship between Ir and various signaling pathways involved in cardiovascular disease. However, Ir may have therapeutic potential in protecting against various cardiovascular diseases through its multiple effects on the above-mentioned pathways.

### 1.3. Function and Effect of FNDC5/Irisin on Cells

Skeletal muscles involved in exercise may have a secretory function, producing myokines. Ir is one of the myokines released into the blood by the muscle fibers under the influence of exercise [18]. In turn, myokines are involved in metabolic changes and participate in the regulation of cardiovascular function [40]. They also interact with various cells and organs [41]. Ir can be found in the cytoplasm and cell membrane in various tissues. It can also be released into the blood. Studies on Ir indicated that it affects various organs, and its effects may be related to organ adaptation to physical exercise. Additionally, studies confirmed the effects of Ir on adipose tissue, heart muscle, bone tissue and angiogenesis.

#### 1.3.1. The Effect of Physical Exertion on the Secretion of Irisin

High-intensity exercise results in a greater increase in Ir levels compared to low-intensity exercise [42]. Studies suggested that Ir levels peaked at 6 h after exercise and then returned to pre-exercise levels within 24 h, contributing to the beneficial effects of exercise [43]. Kraemer et al. [44] and Huh et al. [45] demonstrated a rapid increase in Ir levels in response to aerobic exercise. However, these levels returned to baseline within 60–90 min. Jóźków et al. [46] suggested that extreme acute physical exertion inhibited the release of Ir. Marathon runners experienced a decrease in Ir levels that persisted for 7 days. This finding was supported by a study of Joro et al. [47], in which athletes who underwent overly intense training presented with decreased serum Ir levels compared to those who underwent balanced training. A stable and consistent increase in serum Ir levels is possible after a lifestyle change and consistent year-long training, highlighting the importance of training load and regular physical activity in this process [48]. Increased physical activity may be beneficial in the management of metabolic disorders and cardiovascular disease. A sedentary lifestyle increases the risk of heart disease, whereas regular physical activity reduces this risk [49]. Lecker et al. [50] observed a positive correlation between Ir levels and cardiac exercise capacity.

Regular physical activity is thought to have a positive effect on health, which is supported by Rana et al. [51], who showed that circulating Ir levels were positively correlated with telomere length. Shorter telomeres are associated with cell aging and an increased risk of myocardial infarction [52]. Furthermore, Emanuele et al. [53] suggested that healthy centenarians had higher Ir levels than young individuals and those affected by myocardial infarction.

#### 1.3.2. Impact of FNDC5/Irisin on Adipose Cells

**Figure 2:**
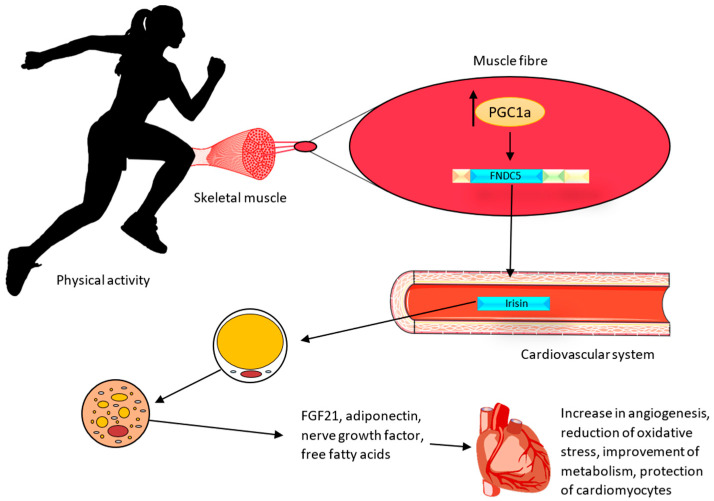
Effect of irisin on the heart muscle after physical activity. During physical activity, muscles release irisin, which affects the browning process of WAT. The protective effect is achieved through the secretion of the fibroblast growth factor (FGF21), adiponectin (exhibiting anti-inflammatory and anti-atherosclerotic effects), nerve growth factors responsible for the regulation of neuronal growth and survival, and free fatty acids [55].

In vivo studies on Ir indicated that it increases the expression of mitochondrial uncoupling protein 1 (UCP1) and the conversion of white adipose tissue into brown adipose tissue in mice [18]. The UCP1 protein, which is an ion channel in the inner membrane of mitochondria, causes the generation of thermal energy at the expense of a decrease in adenosine triphosphate (ATP) production. White adipose tissue (WAT) is commonly found in the human body. It is believed that under the influence of physical activity (Figure 2), with the participation of Ir, WAT can undergo a browning process, thus having the similar characteristics of brown adipose tissue (BAT). This tissue is termed ‘beige adipose tissue’ (bAT) [54]. The browning of WAT can lead to the activation of cardioprotective mechanisms. BAT is responsible for the secretion of biologically active compounds known as batokines. They include the fibroblast growth factor (FGF21), adiponectin, which exhibits anti-inflammatory and anti-atherosclerotic effects, nerve growth factors responsible for the regulation of neuronal growth and survival, and free fatty acids [55]. BAT also regulates vascular tone by secreting vasoconstriction and vasodilation factors from perivascular and epicardial tissues [56]. In addition, BAT decreases triglyceride levels and attenuates insulin resistance [57]. Therefore, the effects of physical exercise and Ir on the adipose tissue may also have a role in preventing cardiovascular disease.

#### 1.3.3. Impact of FNDC5/Irisin on Osteoclasts and Osteoblasts

The action of Ir is also associated with the remodeling and altered metabolism of other tissues involved in exercise. Kim et al. [29] and Greenhill et al. [58] showed that Ir increased the levels of sclerostin involved in the stimulation of bone resorption and remodeling. They also found that Ir affected osteoclast differentiation. Ir can also stimulate the differentiation of cells into mature osteoblasts [59]. In addition, Greenhill et al. [58] showed that blocking integrin αVβ5 inhibited the effect of Ir on osteocytes and adipocytes. Wahab et al. [60] made a similar observation for adipose cells. According to both studies, Ir binds to integrin αVβ5. The above research does not exclude the possibility that Ir could bind to more than one receptor. Further experiments could indicate the existence of other receptors in different tissue and cell types.

#### 1.3.4. Impact of FNDC5/Irisin on Endothelial Cells, VSCMs and Angiogenesis

In addition to its influence on the skeletal system and adipose tissue, the impact of Ir on angiogenesis has also been observed [61]. Angiogenesis, which is associated with tissue repair and restoration, plays an essential role in the therapeutic process when apoptosis of cardiomyocytes occurs. Factors that can stimulate the formation of new blood vessels may be important for effective treatment. Ir was shown to stimulate angiogenesis in both in vitro and in vivo models by increasing migration and the formation of capillary structures [61]. These processes are crucial, and their abnormalities may be associated with the pathology of cardiovascular disease, peripheral artery disease and chronic metabolic diseases. Ischemic heart disease (IHD) is characterized by impaired blood flow due to obstruction of the main coronary arteries. Therefore, potential therapeutic strategies for IHD focus on stimulating the processes of angiogenesis and neovascularization to restore the blood supply to the myocardial tissue [62].

**Figure 3:**
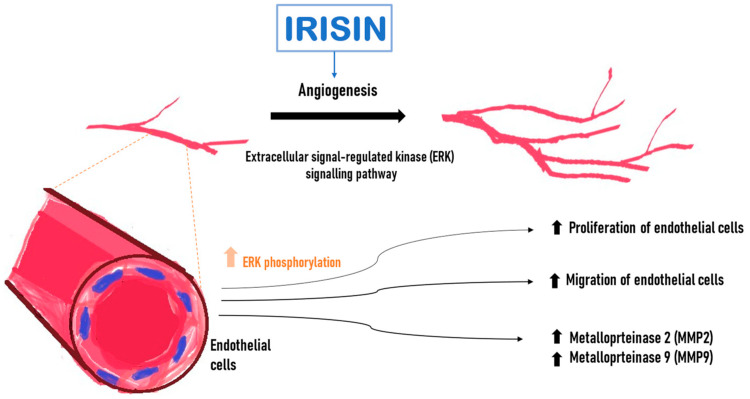
Effect of irisin (Ir) on endothelial cells. Increase in the level of active phosphorylated ERK kinase (P-ERK). Higher Ir levels increase the expression of matrix metalloproteinase 2 (MMP2) and 9 (MMP9) mRNA, promote endothelial cell proliferation and increase endothelial cell migration.

Wu et al. [61] demonstrated an increased proliferation and migration of human endothelial cells isolated from the umbilical vein (HUVEC) after incubation with Ir. Following Ir administration into the medium in which endothelial cells were cultured, it induced a significant increase in capillary-like tube formation after 6 h compared with the control. Ir affected endothelial cell proliferation through the extracellular signal-regulated kinase (ERK) signaling pathway [61]. It was confirmed that phosphorylated ERK (P-ERK) was significantly increased after treatment with Ir for 5 min and 10 min (Figure 3). In addition, incubation with Ir stimulated an increase in metalloproteinase 2 (MMP2) and 9 (MMP9) mRNA expression and their gelatinolytic activity in endothelial cells. MMP2 and MMP9 were emphasized because their type IV collagenase activity is essential during the initial phase of angiogenesis [61].

Altaweel et al. [63] showed that an intraperitoneal injection of Ir in rats increased the thickness of the intima–media complex, the number of smooth muscle cell nuclei and the number of elastic lamellae in the medial layer of the thoracic aorta, which is composed of vascular smooth muscle cells (VSMCs). These cells proliferate and migrate toward the intima, thus contributing to vascular remodeling and lamellar formation. Throughout this process, endothelial cell proliferation acts as a protective mechanism to repair damaged endothelium, helping to maintain endothelial integrity [64]. In circulating extracellular vesicles, Ir mediates the protective effects of physical activity against vascular aging by increasing the stability of SIRT6 in VSMCs in a DnaJ/Hsp40-chaperone-dependent manner [65]. 

#### 1.3.5. Impact of FNDC5/Irisin on Cardiomyocytes

**Figure 4:**
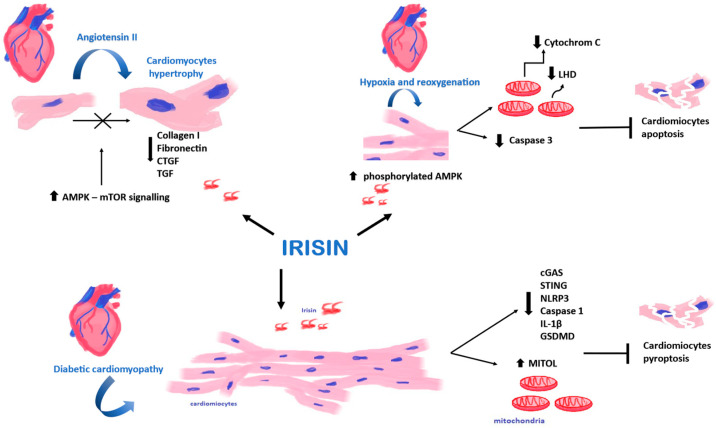
Effect of Irisin (Ir) on cardiomyocytes. Ir affects cardiomyocytes in hypoxia and reoxygenation, affects cardiomyocytes in diabetic cardiomyopathy, and demonstrates the inhibitory effect on cardiomyocyte hypertrophy.

Current studies have demonstrated that Ir is mainly secreted into the bloodstream by cardiomyocytes. In turn, skeletal muscles at rest release smaller amounts of Ir than cardiomyocytes [66]. Yu et al. [27] found that FNDC5/Ir expression was elevated in hypertrophic cardiomyocytes and skeletal muscle cells after the administration of angiotensin II (Ang II). Cardiomyocytes were treated with Ang II to induce hypertrophy. At the same time, cardiomyocytes were treated with Ir or PBS. They found that Ir prevented the development of cardiac hypertrophy by activating the AMPK–mTOR signaling pathway (Figure 4). The AMPK–mTOR axis is an important signaling pathway in the regulation of cardiac hypertrophy [67]. Cardiomyocyte hypertrophy is an early response of the heart to stress that precedes extracellular matrix expansion, interstitial fibrosis and the overt clinical manifestations of heart failure [68]. In addition, the expression of genes responsible for the phenotypic changes (genes encoding collagen I, fibronectin, CTGF and TGF) decreased in Ir-treated cardiomyocytes. However, hypertrophic changes were observed in cardiomyocytes after silencing the expression of the *FNDC5* gene [27].

In turn, Yue et al. [69] found that the pre-treatment of mouse cardiomyocytes with Ir could also reduce ischemia–reperfusion damage to cardiomyocytes. Cardiomyocytes were cultured under conditions of hypoxia and reoxygenation. Their study found that pre-treatment of cardiomyocytes with Ir increased the survival rate of cardiomyocytes, and it also reduced cell apoptosis and caspase 3 activity after transient hypoxia. Additionally, the study found significantly decreased cytochrome c release from mitochondria and lactate dehydrogenase (LHD) into the extracellular space, which could indicate a protective role of Ir [69]. The AMP–protein kinase (AMPK), which is a regulator of energy metabolism, plays an essential role in the protective activity of Ir [70]. It is activated by phosphorylation to protect cardiomyocytes from myocardial ischemia–reperfusion injury by regulating mitochondrial function. Yue et al. [69] found that pre-treatment with Ir increased the level of phosphorylated AMPK. In addition, they showed that pre-treatment with Ir or metformin had the same effect, increasing AMPK activity in cardiomyocytes after hypoxia and reoxygenation.

Also, Fan et al. [71] conducted a study on the rat H9C2 cardiomyocyte cell line under the following conditions: 30 min of hypoxia, 4 h of reoxygenation and high glucose stress. Their study demonstrated the impact of Ir on the activation of the AMPK pathway. The administration of human Ir sustained mitochondrial function through the AMPK pathway and cardiomyocyte survival. Also, Moscoso et al. [72] found that rat H9C2 cardiomyocytes treated with Ir and subjected to hypoxia showed higher survival rates. In turn, in their in vitro study, Xie et al. [73] observed an increased metabolic rate, inhibited proliferation and an enhanced differentiation of H9C2 cells after Ir administration. This suggests the possible use of Ir in the treatment of patients with reperfusion injury. However, further studies are warranted to explain the mechanism of action of Ir on cardiomyocytes.

## 2. Significance of FNDC5/Irisin in Cardiovascular Disease

Blood levels of Ir change in the course of cardiovascular disease. Hirayama et al. [74] showed that Ir levels correlated negatively with lipid levels and positively with the fibrous connective tissue content in the left main coronary artery (LMCA). Patients with the mean Ir levels presented with fewer atherosclerotic lesions than those with higher Ir levels, which suggests that Ir could be a potentially useful biomarker for diagnosing the severity of atherosclerotic lesions in the coronary arteries. In turn, Park et al. [75] found elevated Ir levels in patients with coronary artery disease after percutaneous coronary intervention (PCI), metabolic syndrome, cardiometabolic changes and cardiovascular disease. However, Aronis et al. [76] showed that circulating Ir levels could not be used to predict the development of acute coronary syndromes (ACS) in asymptomatic subjects. In addition, it was found that elevated Ir levels in individuals with established coronary artery disease after PCI were associated with the development of severe cardiovascular events [76].

### 2.1. Significance of FNDC5/Irisin in Myocardial Infarction

Myocardial infarction (MI) occurs due to a lack of blood flow through the coronary arteries, which leads to myocardial ischemia, resulting in the death of cardiomyocytes [77]. Biomarkers used to diagnose MI are given in Table 1.

Cardiac troponin T and I isoforms (cTnT and cTnI) are the most common diagnostic markers for MI that are characterized by high sensitivity. Unfortunately, their levels do not increase in the first hours after the onset of MI. Therefore, the activity of creatine kinase (CK-MB) is also determined [78]. New biomarkers are sought to improve the possibilities of early diagnosis and sequelae of MI. It seems that Ir could be one of such biomarkers.

Aydin et al. [79] showed that Ir levels in the blood decreased after the onset of MI (Figure 3). According to them, as a biomarker, Ir has a diagnostic specificity and sensitivity similar to CK-MB [80]. In addition, Ir serum levels correlate positively with cTnT, cTnI and CK-MB levels. However, correlations of the levels and the relationships of Ir with other MI markers (Table 1) have not been studied [79]. The studies were conducted on a small group of patients. Furthermore, there is a lack of consistency regarding methods to determine Ir levels [81]. Therefore, it seems important to further expand knowledge on the usefulness of assessing serum Ir levels in MI. Interestingly, Matsuo et al. [82] found a release of Ir from damaged cardiomyocytes, which may explain its temporary increase in blood after MI, which was followed by a gradual decrease in its level. Aydin et al. [79] showed that blood Ir levels decreased between 6 and 48 h after the onset of acute MI. then, 72 h after MI, Ir levels gradually returned to pre-MI levels.

During MI, cardiomyocyte metabolism is significantly decreased. As a result of oxygen deprivation, the amount of ATP produced in the respiratory chain decreases. Due to oxygen deprivation, 30 min after the onset of MI, the myocardial ATP level decreases by 50%. ATP in the heart is sufficient to maintain cardiac homeostasis only for a short period of time after MI. To maintain proper cellular function, energy must be obtained from other pathways. Ir affects the expression of thermogenin, which decreases ATP formation in the mitochondrial respiratory chain and dissipates energy in the form of heat. Decreased serum Ir levels may indicate no release of Ir from cardiomyocytes and its involvement in altering the metabolism of these cells during MI [83].

**Figure 5:**
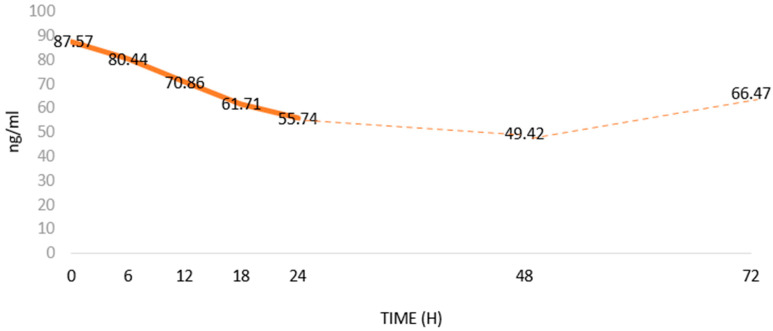
Changes in serum Irisin (Ir) levels in patients with acute myocardial infarction (MI) based on Aydin et al. [79]; changes in Ir levels in ng/mL over time after MI.

Anastasilakis et al. [80] indicated that Ir levels were also related to the degree of coronary stenosis. They observed that Ir secretion was regulated according to the sufficiency of blood supply to the muscle of the heart. Under the reduced supply of oxygenated blood, myocardial cells release less Ir. In turn, Bashar et al. [84] showed a positive correlation between serum Ir levels and acute MI (Figure 5). Higher serum Ir levels decreased the severity of MI in the exercise-trained rats compared to control animals with lower serum Ir levels. Seo et al. [85] showed that Ir levels increased in rats performing treadmill exercise for 12 weeks and also correlated positively with the ejection fraction. This may suggest that a systemic elevation of Ir levels could have a beneficial effect on cardiac function in rats, which was supported by Liao et al. [86], who found that in rats after MI, Ir administration improved angiogenesis in the border zone of MI (the area between normal and post-MI tissues) and decreased cardiomyocyte apoptosis, but it did not influence cardiomyocyte proliferation. The intraperitoneal administration of human exogenous Ir to the zebrafish (Danio rerio) increased diastolic volume, heart rate and cardiac output [87]. The therapeutic administration of Ir may be an effective adjunctive therapy in patients in the future due to its positive effects on cardiomyocyte survival and function.

However, it is believed that long-term increased levels of Ir in the blood may have negative effects. Higher Ir levels increase the production of reactive oxygen species (ROS) and may increase cardiomyocyte apoptosis [88]. During a 3-year follow-up, Hsieh et al. confirmed the effect of high serum Ir levels on the occurrence of cardiovascular events in patients with acute coronary syndrome (ACS) [89]. Patients with significantly higher Ir levels were characterized by higher mortality rates one year after MI, which indicates a link between the risk of death after MI and serum Ir levels [79]. Increased serum Ir levels may also be caused by drug therapy. Sildenafil and iloprost dilate blood vessels and increase blood flow to tissues. After the administration of these agents, Ir levels were restored to the levels before MI [90].

The above reports suggest that changes in serum Ir levels may be important in the course of MI and other cardiovascular diseases. When MI occurs, no release of Ir, or the decreased serum Ir levels may be related to changes in cardiomyocyte metabolism.

### 2.2. Significance of FNDC5/Irisin in Atherosclerosis, Coronary Artery Disease and Hypertension

Atherosclerosis is a chronic inflammatory disease that leads to pathological changes in the structure of the heart. These changes include lipid aggregation, fibrous growth, calcium deposition, degeneration and calcification of the middle layer of the arteries, eventually leading to bleeding, plaque rupture and local thrombosis [91]. Lower levels of Ir are associated with more severe atherosclerotic changes, which may suggest the influence of Ir in delaying the development of atherosclerosis [92]. Ir may influence the inhibition of the progression of atherosclerosis by deactivating ROS–NLRP3 inflammasome signaling [93]. Ir levels correlate with the presence of atherosclerotic plaques in the left main coronary artery (LMCA), which suggests that Ir may also be a potential biomarker useful for the detection of atherosclerotic plaques in coronary vessels [74].

Ir levels are decreased in patients with coronary artery disease [94]. Circulating Ir levels cannot be used to predict the development of acute coronary syndromes (ACS) in healthy individuals. However, an increase in its level in individuals with coronary artery disease after percutaneous coronary intervention is related to the development of major adverse cardiovascular events (MACEs) [76]. Ir levels are lower in patients with atherosclerosis than in healthy individuals [95] and higher in those with hypertension compared to healthy individuals and are also positively correlated with diastolic blood pressure [96].

Ir improves endothelium-dependent vasodilation by activating the AMPK–Akt–eNOS–NO signaling pathway, thereby lowering blood pressure [97]. Ir lowers blood pressure by activating the Nrf2 signaling pathway in the paraventricular nucleus of the hypothalamus [98]. However, the exact mechanisms involved in the different effects of Ir on blood pressure are not known and require further investigation [99]. 

In conclusion, Ir may mediate blood pressure reduction and vasodilation after exercise [100] and support cardiac rehabilitation processes [101]. 

### 2.3. Protective Effect of FNDC5/Irisin on the Cardiovascular System

Studies have demonstrated that Ir may have a protective effect on the cardiovascular system [102]. It is expressed under the influence of PGC-1α, which is involved in mitochondrial biogenesis [10] and the regulation of mitochondrial function through the UCP1 protein [12]. In turn, mitochondrial function is crucial in regulating cardiomyocyte survival during MI [102]. Damaged mitochondria generate ROS in the cell, which can initiate apoptosis [103]. Mitochondria release pro-apoptotic factors to initiate programmed cell death. Therefore, they are key regulators of cell survival. In addition, they are also essential for ATP production that maintains cardiomyocyte contractility and metabolism [104,105].

Xin et al. [102] found that the expression of the *OPA1* gene increased when exogenous Ir was administered to cardiomyocytes under hypoxic conditions. The gene encodes optic atrophy protein 1, which is a dynamin-like GTPase. The OPA1 protein plays a role in apoptosis as well as the maintenance of mitochondrial DNA (mtDNA), and it is also responsible for oxidative phosphorylation in mitochondria. The above studies showed that *OPA1* gene expression decreased under hypoxia or MI. In turn, cardiomyocytes with an overexpression of the *OPA1* gene showed higher survival rates during hypoxia due to the induction of mitophagy, i.e., the selective removal of damaged mitochondria from the cell. Such removal could result in the inhibition of the production of pro-apoptotic factors. It also protects the cells from programmed cell death. In addition, Xin et al. [102] showed that silencing the *OPA1* gene eliminated the cardioprotective effects of Ir. They suggested that the protective effect of Ir after MI was related to mitophagy induced by the increase in *OPA1* expression.

The role of Ir in the protection of the myocardium from ischemic injury has not been fully elucidated. However, studies found that myocardial ischemia stimulated an increase in *FNDC5* gene expression and the release of Ir from cardiomyocytes [106]. Additionally, high levels of Ir decreased the expression of apoptotic proteins, including active caspase 3, PARP and annexin V [107]. Additionally, Ir is also responsible for the increased phosphorylation (activation) of p38 MAPK kinase, which regulates the cell cycle, apoptosis and differentiation, and the expression of superoxide dismutase (SOD)-1, which protects the cell from ROS in the ischemic myocardium, thus contributing to the inhibition of cardiomyocyte apoptosis [108]. In addition, Wang et al. [108] showed that Ir protected mitochondria against ischemia–reperfusion injury through an SOD2-dependent mitochondria mechanism. This is a major mitochondrial antioxidant enzyme regulating ROS production and its levels decrease during cardiac ischemia–reperfusion injury. The reduced SOD-2 activity could be restored by administering Ir to cardiomyocytes [108]. Ir also increased the survival rate of cardiomyocytes exposed to hypoxia and reoxygenation, which was closely related to decreased mitochondrial permeability transition pore (mPTP), mitochondrial swelling and apoptosis [109]. In summary, Ir increases cardiomyocyte survival after hypoxia by maintaining mitochondrial homeostasis and reducing oxidative stress, leading to an inhibition of apoptosis.

In their recent study, Li et al. [110] also showed that Ir prevented autophagy in pressure overload cardiac hypertrophy. It blocked the AMPK–ULK1 pathway of the *mTOR* gene responsible for regulating cell growth, proliferation and movement. This may indicate its potential role in the development of left ventricular (LV) dysfunction and hypertrophy [110].

### 2.4. Potential Therapeutic Use of FNDC5/Irisin in Cardiovascular Disease

Recent studies have indicated the possibility of using Ir in cardiovascular therapy. Its effect on inhibiting the process of cardiomyocyte apoptosis seems to be important. Studies have shown that the administration of exogenous Ir reduced the apoptosis of cardiac muscle cells and decreased oxidative stress. The myocardium of mice treated with Ir showed a significant reduction in cardiomyocyte apoptosis compared to control mice that were not treated with Ir [1]. Zhang et al. [111] found a cardioprotective effect of Ir. They conducted a study on rat H9C2 cardiomyocytes of controls and those with *FNDC5* gene overexpression treated with doxorubicin (DOX). In cardiomyocytes with higher FNDC5 mRNA levels, a reduction in oxidative stress parameters and decreased apoptosis were observed. An increase in serum Ir levels may have a therapeutic value in alleviating cardiotoxicity during chemotherapy with DOX [111].

In turn, Ouyang et al. [112] conducted a study on cardiomyocytes after exposure to lipopolysaccharides (LPS), inducing septic cardiomyopathy. They showed that the combination of Ir and melatonin (a pineal gland hormone with antioxidant effects) ameliorated LPS exposure in cardiomyocytes through suppressing macrophage-stimulating 1 (Mst1), which is activated by LPS. In turn, Mst1 caused an activation of c-Jun N-terminal kinase (JNK), leading to increased oxidative stress parameters, impaired ATP metabolism and reduced mitochondrial membrane potential, which resulted in cardiomyocyte apoptosis. Their study showed that the combination of Ir and melatonin promoted cardiomyocyte survival after exposure to LPS and improved mitochondrial homeostasis. Ouyang et al. suggested that the therapeutic administration of Ir with melatonin after sepsis-induced cardiomyopathy could have a protective effect on the cardiac muscle [112]. In addition, Ir could also reduce angiotensin II-induced cardiomyocyte apoptosis through autophagy and inhibition of the apoptosis signaling factor [113].

Apart from stimulating angiogenesis, the induction of cell proliferation and differentiation is an important part of the repair process. Zhao et al. [114] showed satisfactory effects of regenerative therapy in their in vivo study of the mouse myocardial infarction model using cardiac progenitor cells (COCs). Nkx 2.5^+^ CPC stable cells isolated from mouse embryonic stem cells were treated with Ir for 24 h before transplantation. After inducing MI in mice, the cells were engrafted. After 8 weeks, myocardial functions were assessed by echocardiographic measurements, immunofluorescent staining and immunohistochemistry of CD-31 and Ki-67. Their study found that Ir promoted Nkx 2.5^+^ CPC-induced cardiac regeneration compared to control cells that were not treated with Ir. Ir-induced CPCs improved ventricular function and decreased cardiomyocyte hypertrophy. They also found that the number of CD-31-positive vessels was increased. Additionally, the study found higher Ki-67 expression and an attenuation of myocardial interstitial fibrosis [114]. In addition, Zhao et al. [114] found two important changes increasing the transcriptional activity of specific DNA regions in Ir-treated CPCs. Ir treatment resulted in histone deacetylase 4 (HDAC4) inhibition. Additionally, an activation of acetylated p38 was found following Ir treatment [114], leading to the activation of p38, which is crucial for cardiogenesis [115].

## 3. Conclusions

Previous studies investigating the importance of Ir in MI and other cardiovascular diseases have indicated that the determination of the changes in Ir levels may have a potential diagnostic value. Increasing levels of physical activity may be a preventive measure for cardiovascular disease. However, more detailed research is warranted in this area. In the future, the impact of the protective effect of Ir could be used to design comprehensive cardiac rehabilitation programs. Investigating how exercise affects systemic levels of Ir during rehabilitation would be useful. In addition, research has also suggested the possible use of Ir in cardiac regenerative therapy and in inhibiting cardiomyocyte apoptosis. Determination of Ir levels may also be useful in screening tests of people at risk for cardiovascular disease. The administration of Ir to patients during the treatment of cardiovascular disease could be equally important, as current results in cell lines are promising. Therefore, conducting large clinical trials would be beneficial. However, more precise data are needed on the mechanisms of the impact of Ir on cardiomyocytes, as Ir is still a poorly understood myokine.

## Figures and Tables

**Table 1:** Markers used in the diagnosis of myocardial infarction.

Marker	Marker Detectability	Time of Onset of the Increase (Diagnostic Window)	Time of Maximum Concentration	Time to Normalization
h-FABP	Very early	0.5–1 h	4–10 h	24 h
GP-BB	Very early	1–4 h	6–12 h	24–48 h
Myoglobin	Very early	2–3 h	6–8 h	20–24 h
CK-MB	Early	3.5 h	12–16 h	48–96 h
CK-MB mass	Early	4–5 h	12–16 h	48–96 h
cTnT	Late	4–6 h	12–24 h	7 days
cTnI	Late	4–6 h	12–24 h	10 days

List of abbreviations: heart-type fatty acid-binding protein (h-FABP), glycogen phosphorylase BB (GP-BB), kinase isoenzyme (CK-MB), cardiac troponin T (cTnT), cardiac troponin I (cTnI).
